# Dialysis therapy and mortality in older adults with heart failure and advanced chronic kidney disease: A high-dimensional propensity-matched cohort study

**DOI:** 10.1371/journal.pone.0262706

**Published:** 2022-01-21

**Authors:** Sijie Zheng, Jingrong Yang, Thida C. Tan, Sharina Belani, David Law, Leonid V. Pravoverov, Susan S. Kim, Alan S. Go

**Affiliations:** 1 Department of Nephrology, Kaiser Permanente Oakland Medical Center, Oakland, CA, United States of America; 2 Department of Medicine, University of California, San Francisco, CA, United States of America; 3 Division of Research, Kaiser Permanente Northern California, Oakland, CA, United States of America; 4 Department of Nephrology, Kaiser Permanente San Rafael Medical Center, San Rafael, CA, United States of America; 5 Department of Health System Sciences, Kaiser Permanente Bernard J. Tyson School of Medicine, Pasadena, CA, United States of America; 6 Departments of Epidemiology and Biostatistics, University of California, San Francisco, CA, United States of America; 7 Departments of Medicine, Health Research and Policy, Stanford University School of Medicine, Palo Alto, CA, United States of America; Nazarbayev University School of Medicine, KAZAKHSTAN

## Abstract

**Background:**

Heart failure (HF) and chronic kidney disease (CKD) frequently coexist, and the combination is linked to poor outcomes, but limited data exist to guide optimal management. We evaluated the outcome of dialysis therapy in older patients with HF and advanced CKD.

**Methods:**

We examined adults aged ≥70 years with HF and eGFR ≤20 ml/min/1.73 m^2^ between 2008–2012 and no prior renal replacement therapy, cancer, cirrhosis or organ transplant. We identified patients who initiated chronic dialysis through 2013 and matched patients who did not initiate dialysis on age, gender, diabetes status, being alive on dialysis initiation date, and a high-dimensional propensity score for starting dialysis. Deaths were identified through 2013. We used Cox regression to evaluate the association of chronic dialysis and all-cause death.

**Results:**

Among 348 adults with HF and advanced CKD who initiated dialysis and 947 matched patients who did not start dialysis, mean age was 80±5 years, 51% were women and 33% were Black. The crude rate of death was high overall but lower in those initiating vs. not initiating chronic dialysis (26.1 vs. 32.1 per 100 person-years, respectively, P = 0.02). In multivariable analysis, dialysis was associated with a 33% (95% Confidence Interval:17–46%) lower adjusted rate of death compared with not initiating dialysis.

**Conclusions:**

Among older adults with HF and advanced CKD, dialysis initiation was associated with lower mortality, but absolute rates of death were very high in both groups. Randomized trials should evaluate net outcomes of dialysis vs. conservative management on length and quality of life in this high-risk population.

## Introduction

The number of older adults with advanced chronic kidney disease (CKD) who initiated chronic dialysis (peritoneal dialysis or hemodialysis) has increased significantly over the past several decades, especially among those aged >75 years old [1–3]. While the decision to initiate chronic dialysis is often made for the purpose of prolonging life [3], it is also associated with potential complications, especially in those with a higher comorbidity burden [4,5]. Heart failure (HF) is a large and growing population nationally [6], and CKD is both a frequent comorbid condition affecting >50% of patients and a strong negative prognostic factor for survival [7]. Importantly, there are conflicting data in previous studies about the net clinical outcomes associated with chronic dialysis in the setting of HF and advanced CKD [8–10].

Given the risks associated with dialysis therapy, other options such as conservative CKD management without dialysis may be preferable for patients. Several studies have reported that in older patients, especially those with CKD stage 5, conservative management can be effective in maintaining quality of life [11–13]. However, there have been no randomized controlled trials of chronic dialysis vs. optimal medical management in the growing population of older patients with advanced CKD, and particularly in high-risk CKD patients with HF whose competing risk of short- and long-term mortality can be high [6].

To address this question, we evaluated the association of initiation of chronic dialysis with all-cause mortality in a diverse, high-dimensional propensity-matched cohort of adults with HF and advanced CKD receiving care within an integrated healthcare delivery system.

## Materials and methods

### Source population

The source population was based within Kaiser Permanente Northern California (KPNC), an integrated healthcare delivery system currently providing comprehensive inpatient, emergency and outpatient care for 4.5 million members across 21 hospitals and >255 clinics. KPNC’s membership is highly representative of the local surrounding and statewide population in terms of age, gender, race/ethnicity and socioeconomic status [14]. In addition, nearly all aspects of care are captured through an electronic health record system that is integrated across all practice settings. This study was reviewed by the Kaiser Permanente Northern California Institutional Review Board and determined to be exempt from the requirements for informed consent and Privacy Rule authorization (CN-14-2097-H).

### Study design and sample population

We conducted a retrospective matched cohort study in older adults with HF and advanced CKD between 2008 through 2013 to study the association of initiation of chronic dialysis and mortality. Patients were classified as having HF if they had ≥1 hospitalization with a primary discharge diagnosis of HF (*International Classification of Diseases*, *Ninth Edition* (ICD-9) codes 398.91, 402.01, 402.11, 402.91, 428.0, 428.1, or 428.9) and/or ≥3 outpatient, non-emergency department encounters with a diagnosis of HF with ≥1 of those visits to a cardiologist found in health plan electronic medical records, an approach with a positive predictive value of ≥95% for HF based on review of medical records using Framingham clinical criteria [15]. Advanced CKD was defined as having mean estimated glomerular filtration rate (eGFR) ≤20 ml/min/1.73m^2^ (per the CKD-EPI equation [16]) using the two most recent outpatient serum creatinine measurements within 365 days before the match date.

**Fig 1:**
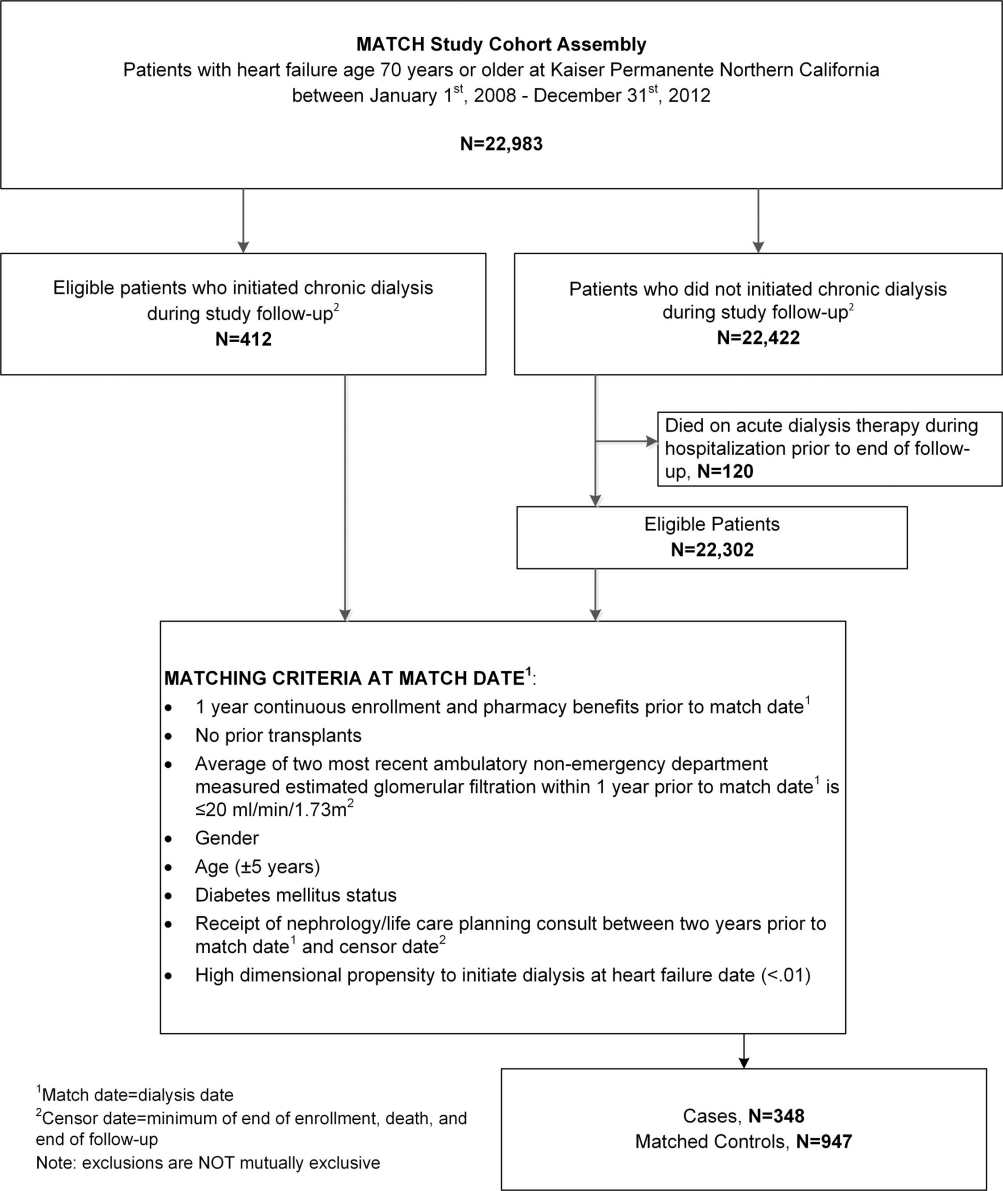
Cohort assembly of matched adults aged ≥70 years old with chronic heart failure and advanced chronic kidney disease between 2006 and 2013.

We next excluded patients who had missing gender, <12 months of continuous health plan membership and drug benefits, prior chronic dialysis, prior organ transplant, diagnosed cirrhosis, or cancer before the match date. We also excluded patients who had <1 day of follow-up due to the end of membership or death, or who developed cirrhosis or cancer during follow-up. In addition, among patients who did not initiate chronic dialysis during follow-up, we excluded anyone who did not survive an episode of acute dialysis therapy during a hospitalization (Fig 1). To generate the matched cohort, we first identified the subset of patients who initiated chronic dialysis (hemodialysis or peritoneal dialysis) using data from a comprehensive health system ESRD Treatment Registry [17]. Patients who initiated chronic dialysis were then matched to patients who did not receive kidney replacement therapy on the calendar date of dialysis initiation using a 1:3 ratio based on the following criteria: age (±5 years), gender, eGFR ≤20 ml/min/1.73m^2^, diabetes mellitus status, receipt of nephrology care or life care planning, and a difference of <0.01 in the propensity to initiate chronic dialysis calculated using a high-dimensional propensity score (hd-PS) [18,19]. To calculate the hd-PS, we performed a logistic regression model to estimate the likelihood of initiating chronic dialysis using 100 empirical covariates that were selected from the top 200 most prevalent variables that differed between exposure groups from each of the following domains: outpatient prescription medications and diagnoses and procedures from inpatient, emergency and outpatient clinic settings. Prioritization of the final variables to include was based on the Bross bias formula.[18]

### Outcomes

Follow-up in the matched cohort occurred through December 31, 2013. Our primary outcome was death from any cause, which was identified from electronic health records (including proxy reports), Social Security vital status files, and California state death certificate data [20,21]. Secondary outcomes in the subset of matched patients who died during follow-up included the location of death and whether palliative care was received based on data ascertained by manual review of electronic health records.

### Covariates

Age, gender, and self-reported race/ethnicity were identified from electronic health records. We ascertained information on coexisting illnesses based on validated algorithms using data on relevant diagnoses or procedures using *International Classification of Diseases*, *Ninth Edition* (ICD-9) and *Current Procedural Terminology* (CPT) codes (codes available on request), laboratory results, or specific therapies from hospitalization, ambulatory visit, laboratory, and pharmacy databases [15,22,23]; as well as a regional diabetes mellitus registry [24]. We ascertained information on quantitative assessments of left ventricular systolic function from the results of echocardiograms, radionuclide scintigraphy, other nuclear imaging modalities, and left ventriculography test results available from health plan imaging databases complemented by manual chart review [7]. Comorbid conditions included cardiovascular conditions and procedures (acute coronary syndrome, coronary artery bypass surgery (CABG), percutaneous coronary intervention (PCI), atrial fibrillation and/or flutter, implantable cardioverter defibrillator, cardiac resynchronization therapy, pacemaker, intracranial hemorrhage, ischemic stroke/transient ischemic attack, peripheral artery disease (PAD), valvular heart disease, ventricular tachycardia or fibrillation), cardiovascular risk factors (tobacco use, diabetes mellitus, hypertension, dyslipidemia), and other non-cardiovascular conditions (cancer, chronic liver disease, chronic lung disease, dementia, depression, thyroid disease, hospitalized bleeding). We also ascertained outpatient visit measures of systolic and diastolic blood pressure, and body mass index (BMI), documented proteinuria (defined as urine dipstick proteinuria of 1+ or greater) [22], as well as outpatient measurements of eGFR, hemoglobin, serum potassium and serum sodium. Targeted medication use was ascertained based on dispensing information from outpatient prescriptions found in health plan pharmacy databases using previously described and validated algorithms and methods [25–27].

### Statistical methods

Analyses were performed using SAS statistical software version 9.4 (Cary, N.C.), with a two-sided P<0.05 considered significant. We compared characteristics between those who did or did not initiate chronic dialysis using Cohen’s D value for continuous variables by taking the standardized difference of means between groups and dividing by the pooled estimate, with a value ≥0.10 considered significant [28,29]; for categorical variables, we used Cramér’s V, with a value ≥0.10 considered significant [30,31]. Crude rates (per 100 person-years) with associated 95% confidence intervals for all-cause death were calculated overall and stratified by initiation of chronic dialysis.

Among matched patients, we next conducted conditional multivariable Cox regression models to evaluate the association of initiation of chronic dialysis and death, with additional adjustment for race, and any covariates that differed at baseline after matching, including age, Hispanic ethnicity, BMI, proteinuria, diagnosed dementia, serum phosphate and sodium levels, treatment with thiazide diuretic, calcium channel blockers, and phosphate binders. Using the final model parameters, we calculated adjusted survival curves associated with initiating or not initiating chronic dialysis therapy. To examine the potential impact of severity of heart failure and socioeconomic status, we performed two sensitively analyses that additionally adjusted for the number of emergency department visits or hospitalizations related to heart failure before baseline and neighborhood-level low educational attainment or annual household income level, and we found no meaningful differences from the main results so only the main results are presented. We handled missing data for continuous variables by creating a categorical variable indicating missingness, and we categorized patients without self-reported race of White, Black, Asian/Pacific Islander, or Native American as “other/unknown.”

For the secondary outcomes, we manually reviewed records of 628 matched patients who died and determined the location of death and whether palliative care was received.

## Results

### Matched cohort assembly and baseline characteristics

Baseline characteristics between unmatched patients aged ≥70 years old with heart failure and advanced CKD who did or did not initiate chronic dialysis are shown in S1 Table. Within this sample, we identified 348 eligible patients who initiated chronic dialysis and 947 matched controls who did not initiate chronic dialysis (using a target 1:3 matching ratio) (Table 1). Overall, in the matched cohort, the mean age was 80.1 years, 56% were women, 31% were persons of color, 15% were Hispanic, and 74% had diabetes. Characteristics were similar between matched patients, except that those who initiated dialysis were more likely to be Hispanic, have prior documented proteinuria; and to be receiving calcium channel blockers, thiazide diuretics, hydralazine, statins or sevelamer. On the other hand, patients who did not initiate dialysis were more likely to have a diagnosed dementia, lower mean systolic blood pressure, lower sodium levels and higher mean hemoglobin and potassium levels at baseline.

**Table 1:** Baseline characteristics of adults aged ≥70 years old with chronic heart failure and advanced chronic kidney disease who initiated chronic dialysis matched to those who did not initiate chronic dialysis between 2008 and 2012.

Characteristic	Overall	Adults with Heart Failure and Advanced CKD Who Initiated Dialysis	Matched Adults with Heart Failure and Advanced CKD Who Did Not Initiate Dialysis	Effect Size
	(N = 1295)	(N = 348)	(N = 947)	
Age, yr, mean (SD)	80 (5)	80 (5)	80 (5)	**0.11**
**Gender, n (%)**				0.00
Women	719 (56)	193 (56)	526 (56)	
Men	576 (44)	155 (44)	421 (44)	
**Race, n (%)**				0.05
White	862 (67)	223 (64)	639 (68)	
Black	130 (10)	37 (11)	93 (10)	
Asian/Pacific Islander	254 (20)	70 (20)	184 (19)	
Native American	18 (1)	7 (2)	11 (1)	
Other/Unknown	31 (2)	11 (3)	20 (2)	
**Known Hispanic ethnicity, n (%)**	199 (15)	76 (22)	123 (13)	**0.11**
**Documented smoking status, n (%)**				
Current or former smoker	769 (59)	212 (61)	557 (59)	0.02
**Cardiovascular history, n (%)**				
Acute coronary syndrome	247 (19)	68 (20)	179 (19)	0.01
Ischemic stroke and/or transient ischemic attack	43 (3)	8 (2)	35 (4)	0.03
Peripheral artery disease	277 (21)	91 (26)	186 (20)	0.07
Mitral and/or aortic valvular disease	249 (19)	68 (20)	181 (19)	0.00
Atrial fibrillation and/or flutter	425 (33)	108 (31)	317 (34)	0.02
**Procedure history, n (%)**				
Coronary artery bypass surgery	41 (3)	5 (1)	36 (4)	0.06
Percutaneous coronary intervention	152 (12)	55 (16)	97 (10)	0.08
Implantable cardioverter defibrillator (ICD)	39 (3)	16 (5)	23 (2)	0.06
Pacemaker	112 (9)	36 (10)	76 (8)	0.04
Cardiac resynchronization therapy	8 (1)	1 (0.3)	7 (0.7)	0.03
**Medical history, n (%)**				
Diabetes mellitus	955 (74)	255 (73)	700 (74)	0.01
Hypertension	1276 (99)	344 (99)	932 (98)	0.02
Diagnosed dementia	88 (67)	8 (2)	80 (8)	**0.11**
Diagnosed depression	236 (18)	50 (14)	186 (20)	0.06
Dyslipidemia	1165 (90)	322 (93)	843 (89)	0.05
Chronic liver disease	43 (3)	11 (3)	32 (3)	0.01
Chronic lung disease	451 (35)	119 (34)	332 (35)	0.01
Hyperthyroidism	67 (5)	25 (7)	42 (4)	0.06
Hypothyroidism	373 (29)	100 (29)	273 (29)	0.00
Extracranial hemorrhage	88 (7)	32 (9)	56 (6)	0.06
**Body mass index, kg/m** ^**2**^ **, n (%)**				**0.11**
≥40.0	54 (4)	9 (3)	45 (5)	
30.0–39.9	372 (29)	97 (28)	275 (29)	
25.0–29.9	435 (34)	120 (35)	315 (33)	
18.5–24.9	394 (30)	117 (34)	277 (29)	
<18.5	12 (0.9)	5 (1)	7 (0.7)	
Unknown	28 (2)	0 (0)	28 (3)	
**Systolic blood pressure category, mmHg, n (%)**				**0.13**
≥180	32 (3)	16 (5)	16 (2)	
160–179	66 (5)	26 (8)	40 (4)	
140–159	233 (18)	66 (19)	167 (18)	
130–139	283 (22)	68 (20)	215 (23)	
121–129	238 (18)	58 (17)	180 (19)	
≤120	439 (34)	114 (32.8)	325 (34.3)	
Unknown	4 (0.3)	0 (0.0)	4 (0.4)	
**Diastolic blood pressure category, mmHg, n (%)**				0.08
≥ 100	2 (0.2)	2 (0.6)	0 (0)	
90–99	17 (1)	6 (12)	11 (1)	
85–89	17 (1)	4 (1)	13 (1)	
81–84	30 (2)	6 (12)	24 (3)	
≤80	1225 (95)	330 (95)	895 (95)	
Missing, n (%)	4 (0.3)	0 (0)	4 (0.4)	
**Baseline medication use, n (%)**				
Alpha blocker	310 (24)	95 (27)	215 (23)	0.05
Angiotensin-converting enzyme inhibitor	247 (19)	66 (19)	181 (19)	0.00
Angiotensin II receptor blocker	254 (20)	65 (19)	189 (20)	0.01
Antiarrhythmic	104 (8)	24 (7)	80 (8)	0.03
Any diuretic	1177 (91)	314 (90)	863 (91)	0.01
Loop	1154 (89)	310 (89)	844 (89)	0.00
Thiazide	215 (17)	89 (26)	126 (13)	**0.15**
Any β-blocker	1074 (83)	300 (86)	774 (82)	0.05
Any aldosterone receptor antagonist	76 (6)	19 (6)	57 (6)	0.01
Isosorbide dinitrate + hydralazine	299 (23)	68 (20)	231 (24)	0.05
Hydralazine	594 (46)	178 (51)	416 (44)	0.06
Nitrate	562 (43)	156 (45)	406 (43)	0.02
Digoxin	88 (7)	22 (6)	66 (7)	0.01
Calcium channel blocker	659 (51)	209 (60)	450 (48)	**0.11**
Statin	1040 (80)	292 (84)	748 (79)	0.05
Other lipid-lowering agent	125 (10)	27 (8)	98 (10)	0.04
Anti-inflammatory drug	19 (2)	4 (1)	15 (2)	0.02
Anti-platelet agent	205 (16)	48 (14)	157 (17)	0.03
Diabetic therapy	608 (47)	149 (43)	459 (49)	0.05
Sevelamer	85 (7)	41 (12)	44 (5)	**0.13**
**Baseline ambulatory, non-emergency department laboratory values**				
Estimated GFR, ml/min/1.73m^2^				
Mean (SD)	15 (4)	12 (4)	17 (3)	**1.41**
Range	4–20	4–20	6–20	
Hemoglobin category, g/dL, n (%)				**0.30**
≥13.0	108 (8)	7 (2)	101 (11)	
12.0–12.9	145 (11)	22 (6)	123 (13)	
11.0–11.9	334 (26)	63 (18)	271 (29)	
10.0–10.9	334 (26)	98 (28)	236 (25)	
9.0–9.9	240 (19)	94 (27)	146 (15)	
<9.0	115 (9)	64 (18)	51 (5)	
Unknown	19 (2)	0 (0)	19 (2)	
Serum sodium, mmol/L				
Mean (SD)	139 (4)	139 (5)	140 (3)	**0.26**
Missing, n (%)	36 (3)	2 (0.6)	34 (4)	
Serum potassium, mmol/L				
Mean (SD)	4.5 (0.6)	4.4 (0.7)	4.5 (0.5)	**0.17**
Missing, n (%)	2 (0.2)	0 (0.0)	2 (0.2)	
Proteinuria, n (%)	839 (65)	292 (84)	547 (58)	**0.24**

### Follow-up and death from any cause

During a total of 2069 person-years of follow-up, only 55 (5%) patients were censored due to disenrollment from the health plan, with median (interquartile range, IQR) follow-up time of 15 (IQR: 6 to 33) months in dialysis initiators and 13 (IQR: 6 to 29) months in matched non-initiators (P = 0.06).

**Fig 2:**
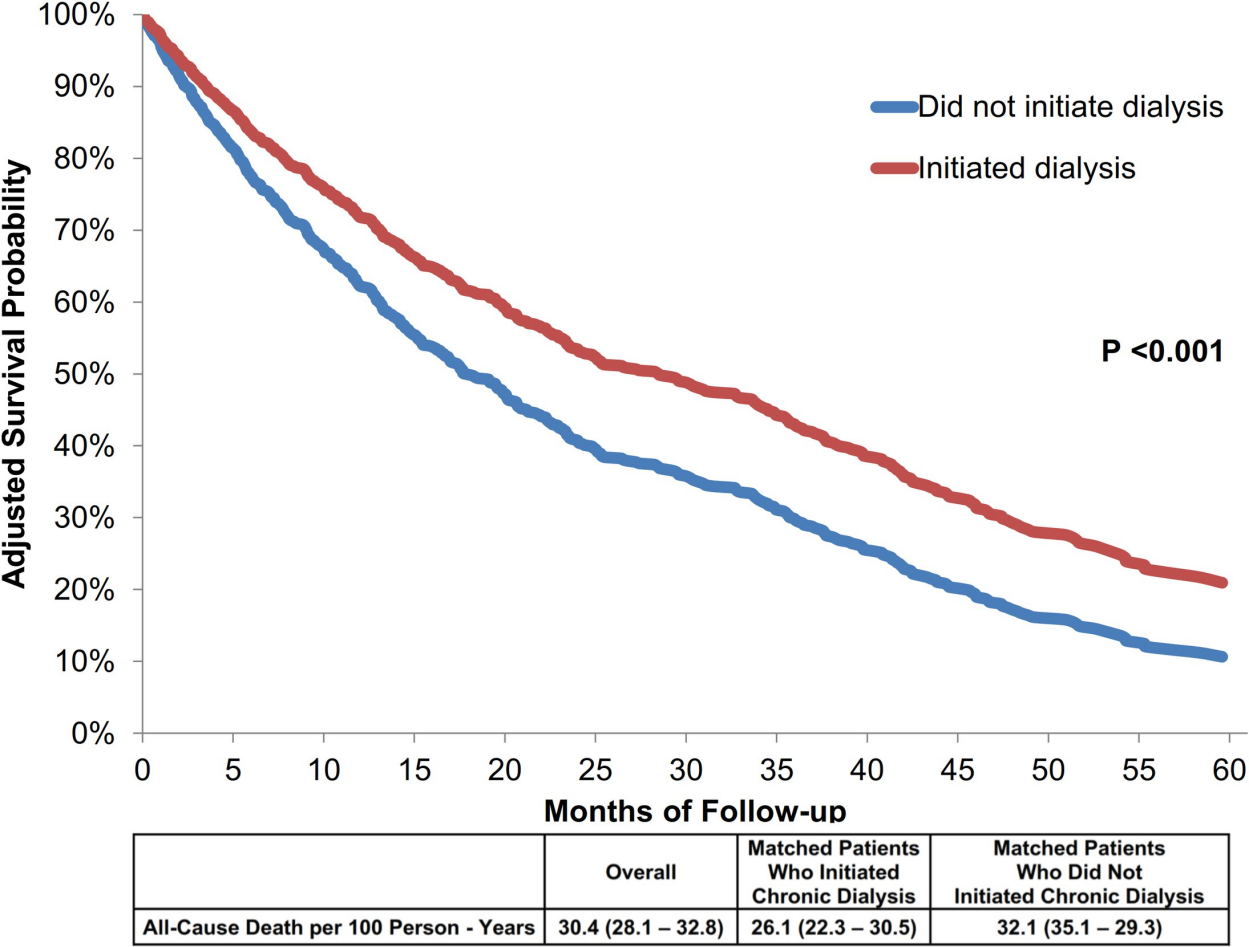
Rate of death and adjusted survival curves of 1,295 matched adults aged ≥70 years old with chronic heart failure and advanced chronic kidney disease between 2008 and 2012. Survival probabilities were calculated for patients with the following characteristics based on the distribution of the overall cohort: 79 years old; male; non-Hispanic white race; with dementia; body mass index <25 kg/m^2^; systolic blood presuure <130 mmHg; hemoglobin ≥12 g/L; potassium 3.5–4.9 mmol/L; sodium 130–139 mmol/L; urine protein dipstick of +1 or greater; receiving diuretic, and calcium channel; and not receiving sevelamer.

Among those who initiated dialysis, 156 (45%) died during follow-up (annual incidence of 26 per 100 person-years) compared to 472 (50%) in those who did not initiate dialysis (annual incidence of 32 per 100 person-years, P = 0.02) (Fig 2). Patients who initiated dialysis were more likely to die in the hospital versus at home compared with those who did not initiate dialysis (53% vs. 26%), and they were also less likely to receive palliative care (Table 2). After additional adjustment for potential confounders, including demographic characteristics, dementia, body mass index, systolic blood pressure, hemoglobin level, potassium level, sodium level, proteinuria, and receipt of calcium channel blockers, thiazide diuretics, and sevelamer, patients that initiated dialysis experienced a lower adjusted rate of death compared with those not receiving dialysis (adjusted hazard ratio 0.67, 95% Confidence Interval:0.54 to 0.83) (Fig 2).

**Table 2:** Location of death and receipt of palliative care among adults ≥70 years old with chronic heart failure and advanced chronic kidney disease between 2008 and 2012 who died during follow-up, overall and stratified by receipt of chronic dialysis.

	Overall (N = 628)	Patients Who Initiated Chronic Dialysis (N = 156)	Patients Who Did Not Initiate Chronic Dialysis (N = 472)	P
**Location of Death**				<0.001
Emergency department/inpatient	204 (33)	82 (53)	122 (26)	
Home	276 (44)	47 (30)	229 (49)	
Other	126 (20)	24 (15)	102 (22)	
Unknown	22 (4)	3 (2)	19 (4)	
**Receipt of Palliative Care**				<0.001
Any	468 (75)	107 (69)	361 (76)	
Inpatient	250 (40)	60 (38)	190 (40)	
Outpatient	213 (34)	46 (29)	167 (35)	
Unspecified	5 (1)	1 (1)	4 (1)	
No	129 (21)	45 (29)	84 (18)	
Unknown	31 (5)	4 (3)	27 (6)	

## Discussion

Patients older than 75 years old are the fastest growing subset of incident ESRD patients nationally, with many having a high comorbidity burden at the time of initiating dialysis [1]. In particular, HF is very frequent among patients receiving chronic dialysis [32]. Among a carefully matched older cohort of adults with HF and advanced CKD (eGFR ≤20 ml/min/1.73m^2^) treated within a fully integrated healthcare delivery system, absolute rates of death were high regardless of the receipt of chronic dialysis. However, we found that initiation of chronic dialysis was independently associated with a modestly improved survival (33% relative reduction), even after matching on key patient features and a high-dimensional propensity score for receiving dialysis, as well as additional statistical adjustment for residual observed differences in baseline patient characteristics and receipt of other therapies. Yet, given the high absolute rates of death in both groups, the longer survival associated with receipt of dialysis was modest, consistent with another study comparing dialysis vs. conservative management in older persons suggesting a benefit of only approximately two months [33]. Of interest, in descriptive analyses among the subgroup of patients who died in our study, those patients initiating dialysis were less likely to die at home or receive palliative care compared with those not receiving dialysis therapy.

Among patients aged ≥65 years initiating chronic dialysis in the U.S., older age is associated with significantly lower survival, with a previous study reporting mean survival of only 16 months for age 80–84 years, 12 months for age 85–89 years and 8 months for age ≥90 years at the time of dialysis initiation [2]. Our study highlights the clinical importance of HF in older adults with advanced CKD, and concomitant HF is a strong negative prognostic factor for death in patients receiving chronic dialysis [8,9]. Among new dialysis initiators, Stack et al. observed higher all-cause mortality in those with CHF regardless of diabetic status [9]. Importantly, even in the absence of CHF, older adults are at risk for loss of independence and reduced functional status and quality of life after dialysis initiation [34,35]. Therefore, in the older adult with HF and advanced CKD, thorough consideration is needed by patients, families and providers about the potential net benefit vs. medical risks and adverse side effects of chronic dialysis therapy before initiation of kidney replacement therapy.

Dialysis is started with the intention of prolonging life and alleviating symptoms. In contrast, initiation of dialysis is frequently associated with a significant decline in functional status in frail patients that appears independent of age, sex, race and functional status before starting dialysis [34]. Thus, the potential survival benefit of dialysis in an older person is countered by the risks of treatment and potential negative impact on quality of life. Furthermore, in certain older patients, dialysis may not improve survival while increasing the risk of functional loss and transfer to a nursing home [36].

There has been increasing recognition that in older persons with advanced CKD and high comorbidity burden, conservative management may be a viable option to kidney replacement therapy [37]. Conservative management can include symptom management, supplemented very low protein diet, dietary potassium restriction, as well as judicious use of diuretics to avoid volume overload [38,39]. In addition, bicarbonate supplementation can be used for the treatment of acidosis and potassium binders for treatment of hyperkalemia. A previous observational study reported that older patients with CKD who were managed conservatively experienced similar survival as patients receiving chronic dialysis [12]. In contrast, several observational studies have reported a favorable association on mortality in older persons with CKD who received dialysis [40–43]. However, the survival advantage associated with dialysis was lower in patients with comorbid cardiovascular conditions [41] and was not observed in persons older than 80 years [44]. The favorable association of dialysis with mortality in the setting of HF may be linked to improved electrolyte and volume control [45], but none of the previous studies have examined survival specifically in patients with advanced CKD and concomitant HF. Collectively, our findings and the existing literature highlight the lack of evidence from randomized controlled trials of conservative management vs. kidney replacement therapy in older patients with CKD complicated by various comorbid conditions in order to more effectively support providers and patients in the shared decision-making process [46].

Our study was strengthened by inclusion of a demographically diverse cohort of older adults with advanced CKD and HF that had comprehensive follow-up on survival for up to five years. We also applied eligibility criteria as well as used statistical methods (i.e., individual matching on patient characteristics and a high-dimensional propensity score to receive dialysis) to identify comparable patients who could be eligible for kidney replacement therapy and did or did not initiate dialysis. Furthermore, we provide relevant information about the location of death and use of palliative care by receipt of dialysis therapy. Our study also had certain limitations. Despite availability of a wide range of available variables and the use of design and statistical techniques to reduce differences between patients, we cannot rule out unmeasured confounding, other treatment selection biases, and variation in patient or caregiver preferences related to kidney replacement therapy.

In conclusion, among older adults with HF and advanced CKD, we found that initiation of chronic dialysis was independently associated with lower all-cause mortality but absolute survival rates were low regardless of receipt of dialysis. Patients initiating dialysis were also less likely to die at home or receive palliative care. Given that therapeutic goals in high-risk older persons with serious morbidity include a balance between maximizing length and quality of life, definitive randomized trials are needed comparing a strategy of initial kidney replacement therapy vs. conservative optimal medical management in older patients with HF and advanced CKD.

## Supporting information

S1 Table Baseline characteristics of adults aged ≥70 years old with chronic heart failure and advanced chronic kidney disease between 2008 and 2012, overall and stratified by receipt of chronic dialysis during follow-up. (DOCX)
