# Supplementary material for: Dialysis therapy and mortality in older adults with heart failure and advanced chronic kidney disease: A high-dimensional propensity-matched cohort study
Source: PLoS One. 2022 Jan 21;17(1):e0262706. doi: 10.1371/journal.pone.0262706 (PMC8782375; doi:10.1371/journal.pone.0262706)
Supplement: S1 Table — (DOCX) [file pone.0262706.s001.docx]

**S1 Table.** **Baseline characteristics of adults aged ≥70 years old with chronic heart failure and advanced chronic kidney disease between 2008 and 2012, overall and stratified by receipt of chronic dialysis during follow-up.**

| **Characteristic** | **Overall** | **Adults with Heart Failure and Advanced CKD Who Initiated Dialysis** | **Adults with Heart Failure and Advanced CKD**  **Who Did Not Initiate Dialysis** | **Effect Size** |
| --- | --- | --- | --- | --- |
|  | **(N=22,714)** | **(N=412)** | **(N=22,302)** |  |
| Age, yr, mean (SD) | 81.4 (6.6) | 77.8 (5.1) | 81.5 (6.6) | **0.62** |
| **Gender, n (%)** |  |  |  | 0.01 |
| Women | 12,142 (53.5) | 208 (50.5) | 11,934 (53.5) |  |
| Men | 10,572 (46.5) | 204 (49.5) | 10,368 (46.5) |  |
| **Race, n (%)** |  |  |  | 0.07 |
| White | 18,293 (80.5) | 258 (62.6) | 18,035 (80.9) |  |
| Black | 1584 (7.0) | 43 (10.4) | 1541 (6.9) |  |
| Asian/Pacific Islander | 2079 (9.2) | 85 (20.6) | 1994 (8.9) |  |
| Native American | 125 (0.6) | 9 (2.2) | 116 (0.5) |  |
| Other/Unknown | 633 (2.8) | 17 (4.1) | 616 (2.8) |  |
| **Known Hispanic ethnicity, n (%)** | 2693 (11.9) | 99 (24.0) | 2594 (11.6) | 0.05 |
| **Documented smoking status, n (%)** |  |  |  |  |
| Current or former smoker | 12,476 (54.9) | 235 (57.0) | 12,241 (54.9) | 0.01 |
| Cardiovascular history, n (%) |  |  |  |  |
| Acute coronary syndrome | 2791 (12.3) | 48 (11.7) | 2743 (12.3) | 0.00 |
| Ischemic stroke and/or transient ischemic attack | 654 (2.9) | 11 (2.7) | 643 (2.9) | 0.00 |
| Intracranial hemorrhage | 279 (1.2) | 4 (1.0) | 275 (1.2) | 0.00 |
| Peripheral artery disease | 1377 (6.1) | 39 (9.5) | 1338 (6.0) | 0.02 |
| Mitral and/or aortic valvular disease | 4786 (21.1) | 59 (14.3) | 4727 (21.2) | 0.02 |
| Atrial fibrillation and/or flutter | 8991 (39.6) | 80 (19.4) | 8911 (40.0) | 0.06 |
| **Procedure history, n (%)** |  |  |  |  |
| Coronary artery bypass surgery | 780 (3.4) | 7 (1.7) | 773 (3.5) | 0.01 |
| Percutaneous coronary intervention | 1918 (8.4) | 35 (8.5) | 1883 (8.4) | 0.00 |
| Implantable cardioverter defibrillator (ICD) | 580 (2.6) | 12 (2.9) | 568 (2.5) | 0.00 |
| Pacemaker | 2007 (8.8) | 24 (5.8) | 1983 (8.9) | 0.01 |
| Cardiac resynchronization therapy | 201 (0.9) | 2 (0.5) | 199 (0.9) | 0.01 |
| **Medical history, n (%)** |  |  |  |  |
| Diabetes mellitus | 8104 (35.7) | 295 (71.6) | 7809 (35.0) | **0.10** |
| Hypertension | 19,590 (86.2) | 404 (98.1) | 19,186 (86.0) | 0.05 |
| Diagnosed dementia | 1643 (7.2) | 3 (0.7) | 1640 (7.4) | 0.03 |
| Diagnosed depression | 3480 (15.3) | 45 (10.9) | 3435 (15.4) | 0.02 |
| Dyslipidemia | 2479 (10.9) | 147 (35.7) | 2332 (10.5) | **0.11** |
| Chronic liver disease | 318 (1.4) | 8 (1.9) | 310 (1.4) | 0.01 |
| Chronic lung disease | 7739 (34.1) | 119 (28.9) | 7620 (34.2) | 0.01 |
| Hyperthyroidism | 786 (3.5) | 22 (5.3) | 764 (3.4) | 0.01 |
| Hypothyroidism | 1119 (4.9) | 39 (9.5) | 1080 (4.8) | 0.03 |
| Extracranial hemorrhage | 1022 (4.5) | 24 (5.8) | 998 (4.5) | 0.01 |
| **Body mass index, kg/m^2^, n (%)** |  |  |  | 0.03 |
| ≥40.0 | 695 (3.1) | 13 (3.2) | 682 (3.1) |  |
| 30.0-39.9 | 6388 (28.1) | 131 (31.8) | 6257 (28.1) |  |
| 25.0-29.9 | 7385 (32.5) | 154 (37.4) | 7231 (32.4) |  |
| 18.5-24.9 | 6816 (30.0) | 103 (25.0) | 6713 (30.1) |  |
| <18.5 | 512 (2.3) | 4 (1.0) | 508 (2.3) |  |
| Unknown | 918 (4.0) | 7 (1.7) | 911 (4.1) |  |
| **Systolic blood pressure category, mmHg, n (%)** |  |  |  | 0.08 |
| ≥180 | 328 (1.4) | 19 (4.6) | 309 (1.4) |  |
| 160-179 | 1010 (4.4) | 49 (11.9) | 961 (4.3) |  |
| 140-159 | 3227 (14.2) | 95 (23.1) | 3132 (14.0) |  |
| 130-139 | 4795 (21.1) | 79 (19.2) | 4716 (21.1) |  |
| 121-129 | 4704 (20.7) | 78 (18.9) | 4626 (20.7) |  |
| ≤120 | 8299 (36.5) | 90 (21.8) | 8209 (36.8) |  |
| Unknown | 351 (1.5) | 2 (0.5) | 349 (1.6) |  |
| **Diastolic blood pressure category, mmHg, n (%)** |  |  |  | 0.02 |
| ≥ 110 | 27 (0.1) | 0 (0.0) | 27 (0.1) |  |
| 100-109 | 109 (0.5) | 2 (0.5) | 107 (0.5) |  |
| 90-99 | 511 (2.2) | 13 (3.2) | 498 (2.2) |  |
| 85-89 | 794 (3.5) | 10 (2.4) | 784 (3.5) |  |
| 81-84 | 1101 (4.8) | 16 (3.9) | 1085 (4.9) |  |
| ≤80 | 19,821 (87.3) | 369 (89.6) | 19,452 (87.2) |  |
| Missing, n (%) | 351 (1.5) | 2 (0.5) | 349 (1.6) |  |
| **Baseline medication use, n (%)** |  |  |  |  |
| Alpha blocker | 75 (0.3) | 36 (8.7) | 39 (0.2) | **0.20** |
| Angiotensin-converting enzyme inhibitor | 86 (0.4) | 44 (10.7) | 42 (0.2) | **0.23** |
| Angiotensin II receptor blocker | 68 (0.3) | 25 (6.1) | 43 (0.2) | **0.14** |
| Antiarrhythmic | 18 (0.1) | 9 (2.2) | 9 (0.0) | **0.10** |
| Any diuretic | 243 (1.1) | 100 (24.3) | 143 (0.6) | **0.31** |
| Loop | 229 (1.0) | 95 (23.1) | 134 (0.6) | **0.30** |
| Thiazide | 55 (0.2) | 20 (4.9) | 35 (0.2) | **0.13** |
| Any β-blocker | 242 (1.1) | 104 (25.2) | 138 (0.6) | **0.32** |
| Any aldosterone receptor antagonist | 13 (0.1) | 5 (1.2) | 8 (0.0) | 0.07 |
| Isosorbide dinitrate + hydralazine | 33 (0.1) | 7 (1.7) | 26 (0.1) | 0.06 |
| Hydralazine | 91 (0.4) | 37 (9.0) | 54 (0.2) | **0.18** |
| Nitrate | 93 (0.4) | 29 (7.0) | 64 (0.3) | **0.14** |
| Digoxin | 19 (0.1) | 9 (2.2) | 10 (0.0) | **0.10** |
| Calcium channel blocker | 179 (0.8) | 84 (20.4) | 95 (0.4) | **0.30** |
| Statin | 234 (1.0) | 102 (24.8) | 132 (0.6) | **0.32** |
| Other lipid-lowering agent | 27 (0.1) | 10 (2.4) | 17 (0.1) | 0.09 |
| Anti-inflammatory drug | 18 (0.1) | 5 (1.2) | 13 (0.1) | 0.05 |
| Anti-platelet agent | 53 (0.2) | 20 (4.9) | 33 (0.1) | **0.13** |
| Diabetic therapy | 130 (0.6) | 57 (13.8) | 73 (0.3) | **0.24** |
| Sevelamer | 11 (0.0) | 6 (1.5) | 5 (0.0) | 0.09 |
| **Baseline ambulatory, non-emergency department laboratory values** |  |  |  |  |
| Estimated GFR, ml/min/1.73m^2^ | 54.5 (18.8) | 23.9 (12.1) | 55.1 (18.4) | **2.00** |
| Mean (SD) | 4.4-131.7 | 5.2-83.4 | 4.4-131.7 |  |
| Range |  |  |  |  |
| Hemoglobin category, g/dL, n (%) |  |  |  | **0.13** |
| ≥13.0 | 8396 (37.0) | 49 (11.9) | 8347 (37.4) |  |
| 12.0-12.9 | 4272 (18.8) | 66 (16.0) | 4206 (18.9) |  |
| 11.0-11.9 | 3508 (15.4) | 91 (22.1) | 3417 (15.3) |  |
| 10.0-10.9 | 1947 (8.6) | 108 (26.2) | 1839 (8.2) |  |
| 9.0-9.9 | 861 (3.8) | 40 (9.7) | 821 (3.7) |  |
| <9.0 | 390 (1.7) | 33 (8.0) | 357 (1.6) |  |
| Unknown | 3340 (14.7) | 25 (6.1) | 3315 (14.9) |  |
| Serum sodium, mmol/L |  |  |  |  |
| Mean (SD) | 139.6 (3.9) | 140.0 (4.0) | 139.6 (3.9) | **0.12** |
| Missing, n (%) | 4503 (19.8) | 35 (8.5) | 4468 (20.0) |  |
| Serum potassium, mmol/L |  |  |  |  |
| Mean (SD) | 4.4 (0.5) | 4.6 (0.6) | 4.4 (0.5) | **0.32** |
| Missing, n (%) | 1971 (8.7) | 12 (2.9) | 1959 (8.8) |  |
| Proteinuria, n (%) | 5589 (24.6) | 307 (74.5) | 5282 (23.7) | **0.16** |
